# MicroRNA miR-263b-5p Regulates Developmental Growth and Cell Association by Suppressing *Laminin A* in *Drosophila*

**DOI:** 10.3390/biology12081096

**Published:** 2023-08-07

**Authors:** Chae Jeong Kim, Hyun Ho Kim, Hee Kyung Kim, Sojeong Lee, Daegyu Jang, Chanhyeok Kim, Do-Hwan Lim

**Affiliations:** School of Systems Biomedical Science, Soongsil University, Seoul 06978, Republic of Korea; jinrourin313@naver.com (C.J.K.); os96kjh123@naver.com (H.H.K.); gmlruddl200@naver.com (H.K.K.); lsj975@naver.com (S.L.); sjj609349@gmail.com (D.J.); chij2001@naver.com (C.K.)

**Keywords:** *Drosophila*, miR-263b, *Laminin A*, extracellular matrix, basement membrane, tissue remodeling, fat body

## Abstract

**Simple Summary:**

The basement membrane (BM) plays a crucial role in various biological processes as a thin layer of the extracellular matrix located at the basal surface of tissues. BMs are regulated by physiological conditions that are altered during development. Specifically, tissue remodeling during metamorphosis in Ecdysozoa is closely associated with BM degradation. However, the mechanisms underlying the regulation of the BM remain unclear. Here, we revealed that the upregulation of the metamorphosis-related microRNA-263b inhibits cell association in the larval fat body by suppressing the expression of *Laminin A*, which is a major component of the BM. This regulatory mechanism is linked to the developmental growth in *Drosophila*. Overall, our results provide valuable insights into the regulation of tissue remodeling and growth during metamorphosis.

**Abstract:**

Basement membranes (BMs) play important roles under various physiological conditions in animals, including ecdysozoans. During development, BMs undergo alterations through diverse intrinsic and extrinsic regulatory mechanisms; however, the full complement of pathways controlling these changes remain unclear. Here, we found that fat body-overexpression of *Drosophila miR-263b*, which is highly expressed during the larval-to-pupal transition, resulted in a decrease in the overall size of the larval fat body, and ultimately, in a severe growth defect accompanied by a reduction in cell proliferation and cell size. Interestingly, we further observed that a large proportion of the larval fat body cells were prematurely disassociated from each other. Moreover, we present evidence that miR-263b-5p suppresses the main component of BMs, *Laminin A* (*LanA*). Through experiments using RNA interference (RNAi) of *LanA*, we found that its depletion phenocopied the effects in *miR-263b*-overexpressing flies. Overall, our findings suggest a potential role for miR-263b in developmental growth and cell association by suppressing *LanA* expression in the *Drosophila* fat body.

## 1. Introduction

Basement membranes (BMs) are specialized thin layers of the extracellular matrix (ECM) found on the basal surface of various tissues [1,2]. These BMs are essential for the growth, organization, maintenance, and function of tissues [1,2]. BMs are composed of different proteins depending on the tissue and developmental stage. Although over 100 proteins associated with BMs have been identified through proteome analysis [3], BMs are primarily composed of two different structured networks made up of either heterotrimeric Laminin or type IV collagen, which are connected by Nidogen (Ndg), Perlecan, and Collagen XV/XVIII homologs [4,5]. These ECM proteins are highly conserved across species [6], but the number of genes encoding each component varies depending on the species. For example, *Drosophila* has one Col IV, one Ndg, and four distinct Laminins, whereas humans have three Col IVs, sixteen Laminins, and two Ndgs [2]. Laminins are a family of heterotrimeric glycoproteins composed of three chains, α, β, and γ [7], and are commonly found in the tissues of most multicellular metazoans [8]. In the *Drosophila* genome, four genes *Laminin A* (*LanA)*, *wing blister* (*wb*), *Laminin B1* (*LanB1*), and *Laminin B2 (LanB2*) encode these three subunits. *Lan A* and *wb* encode two different α subunits, and *LanB1* and *B2* encode the β and γ subunits, respectively [2,7]. As described previously, loss of *LanA* leads to embryonic lethality with severe defects in the muscles, dorsal vessels, and endoderm [9,10]. A null mutation in *LanB1* restricted development until the end of embryogenesis [8].

Tissue remodeling can occur in animals under specific conditions. In particular, holometabolous insects such as *Drosophila* undergo drastic changes via tissue remodeling during the larval-to-pupal transition and metamorphosis. Tissue remodeling is typically accompanied by the deconstruction of tissue organization, such as the loss of cell–cell junctions, cell–BM junctions, and BM components. Various proteases such as matrix metalloproteinases (MMPs) are involved in the degradation of tissue organization [11]. For example, MMP1 is responsible for destroying cell–cell junctions and MMP2 is involved in the degradation of BM components [12]. During metamorphosis, many tissues undergo tissue dissociation, and the larval fat body is a well-known organ that undergoes tissue remodeling in most insects, including *Drosophila* [13]. The BM-associated larval fat body undergoes a gradual remodeling process in which it changes from a well-organized single-cell layer of attached polygonal cells to individual spherical cells. This process results in the redistribution of fat cells across the whole body of the pupa [13]. Distributed larval fat cells are likely to serve as a source of metabolic reserves in pupae and adult flies. However, our understanding of the regulation of this process during development remains limited. 

MicroRNAs (miRNAs) are conserved small non-coding RNAs (ncRNAs) that are approximately 21 nucleotides long and can regulate gene expression through post-transcriptional regulatory mechanisms, such as degradation of mRNA transcripts and/or inhibition of translation [14,15]. These miRNAs are produced through the following two-step process: the transcribed primary miRNAs are first processed into precursor miRNAs by Drosha, and then the precursor miRNAs are matured into miRNA duplexes by Dicer-1, particularly in *Drosophila* [16,17]. Only one strand of the miRNA duplex is associated with the Argonaute-1 (Ago1) protein, which in turn binds to the 3′-UTR of target mRNAs to suppress their expression [18,19,20]. Based on this regulatory framework, miRNAs play a critical role as gene regulators under various physiological conditions. Therefore, many groups have attempted to reveal the regulatory networks of individual miRNAs in various species using different technologies, such as traditional biochemical approaches and high-throughput sequencing technologies, for example, cross-linking immunoprecipitation (CLIP) sequencing. However, a comprehensive understanding of miRNA function is still limited. 

According to several reports, *Drosophila miR-263b* seems to have multiple functions. For example, it has been shown to regulate circadian rhythms and structural plasticity of ventral lateral neurons by targeting *Beadex* (*Bx*) [21] and plays a crucial role in protecting mechanosensory bristles from cell death by suppressing the pro-apoptotic gene *head involution defective* (*hid*) in the retina [22]. Here, we revealed that overexpression of *miR-263b* leads to a reduction of *Drosophila* growth and dissociation of fat body cells by suppressing the BM-related central protein LanA. Overall, our results contribute to a deeper understanding of the role of the interaction of miR-263b-5p and *LanA* in the regulation of BMs during *Drosophila* development.

## 2. Materials and Methods

### 2.1. Drosophila

Flies were reared on standard cornmeal/agar media under noncrowded conditions at 25 °C. The *UAS/GAL4* system was used to overexpress miRNA or knockdown protein coding gene at 25 °C or 29 °C. The following fly lines used in this study were obtained from Bloomington *Drosophila* Stock Center (BDSC): *Cg-GAL4* (BDSC #7011), *UAS-LUC-miR-263b* (BDSC #41146), *UAS-LanA-RNAi^TRiP^* (BDSC #28071), and *w^1118^* (BDSC #5905). The latter strain was used as the control.

### 2.2. Determination of mRNA and miRNA Expression

RNA was isolated from developmental stages, *Drosophila* S2 cells, or larval fat bodies using TRIzol reagent (Invitrogen, Waltham, MA, USA), according to the manufacturer’s instructions. For mRNA transcript levels, reverse transcription quantitative PCR (RT-qPCR) was performed as previously described [23]. In brief, DNA contaminants in the isolated RNA were removed by DNase I treatment, and the RNA was reverse-transcribed using M-MLV reverse transcriptase and random hexamers (Enzynomics, Daejeon, Republic of Korea). For mature miRNA levels, a PCR amplification-based method was used as described previously [24]. Mature miRNA was polyadenylated using *E. coli* poly(A) polymerase (Enzynomics) and reverse-transcribed using M-MLV reverse transcriptase and random hexamers (Enzynomics). Quantitative PCR was performed on a QuantStudio 3 Real-Time PCR instrument (Thermo Fisher Scientific, Waltham, MA, USA) using a small RNA-specific primer and a universal primer set for miRNA, and a gene-specific primer set for mRNA. The primer sequences are listed in Supplementary Table S1.

### 2.3. The Images of Larvae/Flies and Measurement of Body Weight

For the images of larvae and adult flies, the indicated larvae or flies were reared at 29 °C. Images of larvae 5 days after egg laying (AEL) or 3–5 day-old flies of each genotype were captured using a stereomicroscope (Olympus, Shinjuku-ku, Tokyo, Japan). To analyze the body weight of each genotype, ten male or female adult flies (3–5 days old) were transferred to a 1.5 mL tube and weighed using an analytical balance (Mettler Toledo, Columbus, OH, USA).

### 2.4. Analysis of Eclosion Rate

After collecting eggs on standard cornmeal/agar media, three vials containing the appropriate number of eggs for each genotype were placed in a 29 °C incubator. The total number of pupae and empty puparia in each vial was counted at 15 days AEL to allow enough time for eclosion from pupae to adult flies. Eclosion rate was calculated by dividing the number of empty puparia by the total number of pupae.

### 2.5. Wing Measurement

Left wings were taken from ten female flies of each genotype (3–5 days old). The wings were imaged using a stereomicroscope (Olympus, Shinjuku, Tokyo, Japan), and the relative wing size was analyzed using ImageJ [25]. The size of the wing cells was evaluated as previously described [26,27]. Briefly, we counted the number of trichomes, which represent individual wing cells within the standard area (50 × 50 px) of the posterior compartment of the wing. The standard area used for counting was of identical size and located in the same position on the wing. The total cell number of the wings was estimated by dividing the wing size by the evaluated wing cell size.

### 2.6. Analysis of the Larval Fat Body

Fat bodies were dissected from wandering third-instar larvae of the indicated genotypes in cold PBS. To compare the overall size of the fat bodies, dissected larval fat bodies (*n* = 5) were imaged using a stereomicroscope (Olympus).

For phalloidin staining, the fat bodies were fixed with 4% paraformaldehyde (Electron Microscopy Sciences, Hatfield, PA, USA), washed with PBS containing 0.2% Triton X-100, and then stained with Alexa-Fluor 568-phalloidin (1:300; Molecular Probes, Eugene, OR, USA). After additional washing, the stained fat bodies were transferred to mounting medium containing 4′,6-diamidino-2-phenylindole (DAPI; Abcam, Cambridge, UK) and photographed using a confocal laser microscope (Carl Zeiss, Oberkochen, Germany).

### 2.7. The Kyoto Encyclopedia of Genes and Genomes (KEGG) Pathway Enrichment Analysis

The potential target genes of miR-263b-5p were obtained from TargetScanfly [28,29]. Functional enrichment analysis was performed on potential target genes using DAVID [30,31]. The enriched KEGG pathways were determined based on a statistical significance of *p* < 0.05, for each category. Data were visualized using the clusterProfiler package in R software [32].

### 2.8. Luciferase Reporter Assay

For the miR-263b expressing construct, a DNA fragment containing the miR-263b precursor was amplified using the Phusion high-fidelity DNA polymerase (Thermo Fisher Scientific, Waltham, MA, USA) ). The PCR product was inserted into the pMT/V5-His A vector. For the dual-luciferase reporter construct, a DNA fragment with the 3′-UTR of *Lan A* was prepared by PCR amplification and cloned into downstream of *Renilla* luciferase in the psiCHECK-2 vector (Promega, Madison, WI, USA), which expresses both the *Renilla* and firefly luciferases. The primer sets used for plasmid construction are listed in Supplementary Table S1. Site-directed mutagenesis was performed at the miR-263b-5p recognition site in the *Lan A* 3′-UTR using Phusion high-fidelity DNA polymerase and a pair of mutagenic primers (Supplementary Table S1), as previously described [33]. For the reporter assay, the pMT-miR-263b vector (or pMT vector) and psiCHECK-2 with the wild type or mutated 3′-UTR of *LanA* were co-transfected into S2 cells using *Trans*IT^®^-Insect Transfection Reagent (Mirus Bio, Madison, WI, USA). To induce miRNA expression, CuSO_4_ was added to S2 cells the day after transfection at a final concentration of 0.7 mM. A dual-luciferase assay was performed using the dual-luciferase reporter assay system (Promega) 48 h after induction. Firefly luciferase activity was used to normalize the *Renilla* luciferase activity.

## 3. Results

### 3.1. Overexpression of miR-263b in the Fat Body Leads to Defects in the Normal Growth of Drosophila

**Figure 1:**
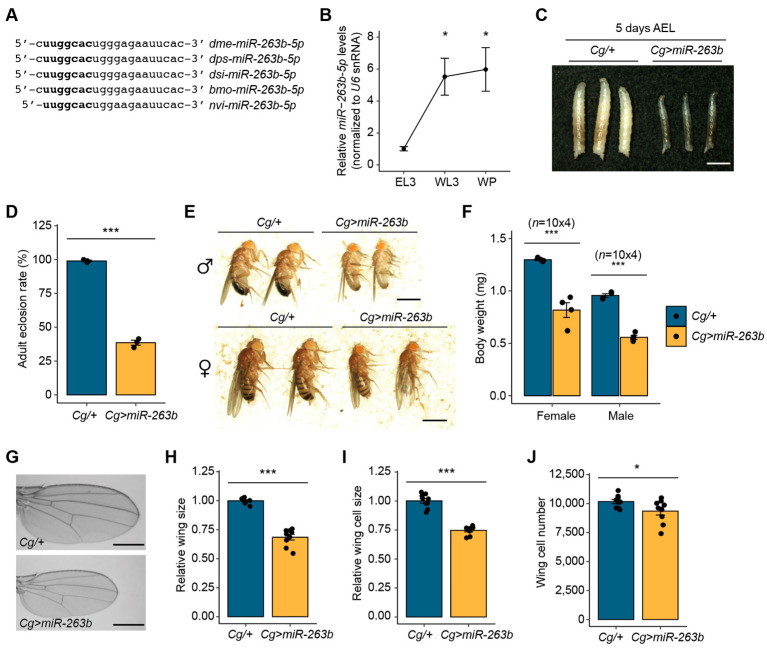
Fat body overexpression of *miR-263b* results in growth reduction in *Drosophila*. (**A**) Multiple sequence alignment with miR-263b-5p in various holometabolous insects. The seed sequence is highlighted in bold. *dme*, *Drosophila melanogaster*; *dps*, *Drosophila pseudoobscura*; *dsi*, *Drosophila simulans*; *bmo*, *Bombyx mori*; *nvi*, *Nasonia vitripennis*. (**B**) Expression levels of *miR-263b-5p* in the fat body at the early third-instar larval (EL3), wandering third-instar larval (WL3), and white pupal (WP) developmental stages. *U6* snRNA was used as an internal control. The line plot is shown as mean ± standard error of the mean (SEM). (**C**) Representative images of larvae of the indicated genotype, 5 days after egg laying (AEL). Scale bar, 1 mm. (**D**) Reduction in the eclosion rate of the pupal-to-adult fly of *Cg>miR-263b*. (**E**) Representative images of male and female flies of *Cg>miR-263b* (3–5 days old). Scale bar, 1 mm. (**F**) Body weight of male and female of each genotype (*n* = 10 flies × 4 tubes). (**G**) Representative wing images of adult female flies of the indicated genotype. Scale bar, 0.5 mm. (**H**) Relative wing size of *Cg>miR-263b* adult female flies (*n* = 9 wings). (**I**) Relative wing cell size of *Cg>miR-263b* adult female flies (*n* = 9 wings). (**J**) Total wing cell number of *Cg>miR-263b* adult female flies. *Cg/+* flies were used as the control. All bar plots (**D**,**F**,**H**,**I**,**J**) are shown as the mean ± SEM. * *p* < 0.05 and *** *p* < 0.001 compared with control, as assessed by Student’s *t*-test.

In our previous study, we found that miR-263b-5p is upregulated during the larval-to-pupal transition in *Drosophila* [34]. Based on these findings, we investigated its biological role. The main strand of miR-263b is highly conserved among the holometabolous insects that undergo metamorphosis, including several *Drosophila* species (Figure 1A). 

To explore the biological functions of miR-263b-5p in the fat body, the primary tissue responsible for energy metabolism, during the larval-to-pupal transition, we first examined the expression levels of *miR-263b-5p* from the early third-instar larval (EL3) stage to the white pupal (WP) stage. Consistent with the expression pattern observed in the whole body [34], the expression of *miR-263b-5p* dramatically increased in the fat body during the larval-to-pupal transition (Figure 1B). These findings suggest a potential role for miR-263b-5p in fat body development during metamorphosis. To regulate miRNA expression in a tissue-specific manner, we used the *UAS/GAL4* system. Using the fat body active *GAL4* driver, *Cg-GAL4* (also active in hemocytes and the lymph gland) [35], we overexpressed *miR-263b* in the fat body. Interestingly, under non-crowded conditions at 25 °C, we observed that the larvae (5 days AEL) with *miR-263b* driven by *Cg-GAL4* (*Cg>miR-263b*) were notably smaller than the control larvae (Figure 1C). We tracked the development of *Cg>miR-263b* to investigate additional phenotypic changes. The overexpression of *miR-263b* in the fat body resulted in a significant decrease in the adult eclosion rate from the pupal case, with a reduction of 61.4% compared to the *Cg/+* control pupae (Figure 1D). Although only a subset of *Cg>miR-263b* pupae successfully emerged in adult flies, *Cg>miR-263b* flies displayed a notable decrease in size compared to control flies of both sexes (Figure 1E). Correspondingly, the body weights of female and male *Cg>miR-263b* flies were dramatically reduced by 63.0 and 58.3%, respectively, compared with the control flies (Figure 1F). These results suggest that miR-263b upregulated in the fat body plays a negative role in normal growth of *Drosophila*.

To further investigate whether the smaller body size of *Cg>miR-263b* flies was attributable to changes in cell number and/or cell size, we analyzed the wings of *Cg>miR-263b* flies as a representative tissue of adult flies. Consistent with the observed body size reduction, we found a significant decrease in wing size in *Cg>miR-263b* female flies compared to controls (Figure 1G,H). Subsequently, we measured the size and number of wing cells in *Cg>miR-263b* female flies. The cell size of the *Cg>miR-263b* female wings was remarkably reduced (Figure 1I), and the total cell number of the *Cg>miR-263b* female wings was slightly lower than that of the *Cg/+* wings (Figure 1J). These observations indicate that the smaller body size of *Cg>miR-263b* adults is the result of a reduction in both cell size and cell number. 

### 3.2. Overexpression of miR-263b Results in Cell Dissociation in the Fat Body

**Figure 2:**
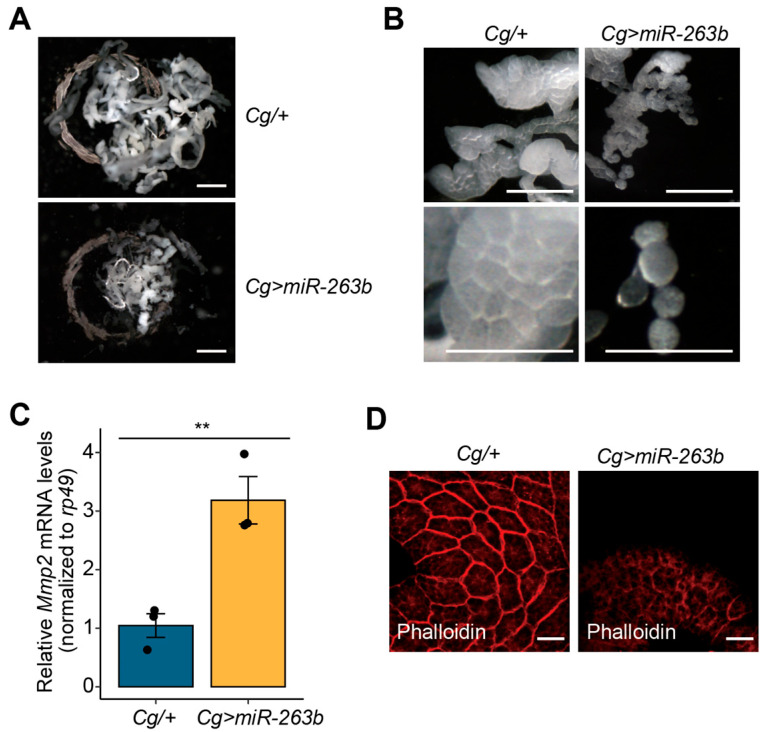
*miR-263b* overexpression results in cell disassociation in the fat body. (**A**) The overall size of the fat bodies dissected from wandering third-instar larvae of each genotype (*n* = 5). Scale bar, 1 mm. (**B**) Magnified images of the fat bodies from wandering third-instar larvae of each genotype. Scale bar, 0.5 mm. (**C**) The expression level of *Mmp2* mRNA transcript in the fat body of *Cg>miR-263b* larvae. *rp49* serves as an internal control. Bar plot is shown as the mean ± SEM. ** *p* < 0.01 compared with control, as assessed by Student’s *t*-test. (**D**) Reduction of F-actin (red) in the fat body of *Cg>miR-263b* larvae. Scale bar, 100 µm.

Since the fat body is a central tissue for energy metabolism associated with developmental growth [36], we next investigated the phenotypic effects of *miR-263b* overexpression in larval fat bodies. To this end, we first analyzed fat body development in *Cg>miR-263b* larvae at 5 days AEL using a stereomicroscope. The overall size of fat bodies extracted from *Cg>miR-263b* larvae was much smaller than that of the control larvae (Figure 2A). This finding supports the possibility that a defect in larval fat body development of *Cg>miR-263b* contributes to a reduction in normal growth.

To determine the cause of this developmental defect in the fat body, we carefully analyzed external changes in the fat bodies of *Cg>miR-263b* larvae and found that they were well-structured and composed of thin layers of tightly conjugated polygonal cells at 5 days AEL. Interestingly, most cells in the fat bodies of *Cg>miR-263b* larvae were physically dissociated from each other and exhibited a spherical shape under identical experimental conditions to that of cells from fat bodies of the *Cg/+* larvae (Figure 2B). These findings suggest that miR-263b negatively regulates cell association in the fat body.

These observations are similar to those observed when the BM is destroyed by MMP2 overexpression during the process of fat body remodeling that occurs during metamorphosis [12,37]. Therefore, we determined whether the expression of *Mmp2* is altered in the fat body upon *miR-263b* overexpression. As expected, the expression of *Mmp2* mRNA transcripts was significantly upregulated in the fat body of *Cg>miR-263b* larvae compared to that in control larvae (Figure 2C). In addition, because alterations in the actin cytoskeleton can contribute to changes in cell shape [38], we examined the expression of F-actin, a key component of the cytoskeleton, in the fat body using phalloidin staining. An F-actin signal was clearly detected in *Cg/+* fat bodies. In contrast, F-actin expression was remarkably diminished in fat bodies of *Cg>miR-263b* larvae, even in those that had not yet undergone dissociation (Figure 2D). Collectively, these observations suggest that the untimely expression of miR-263b leads to the premature dissociation of fat body cells.

### 3.3. miR-263b-5p Suppresses the Expression of Drosophila Laminin A

**Figure 3:**
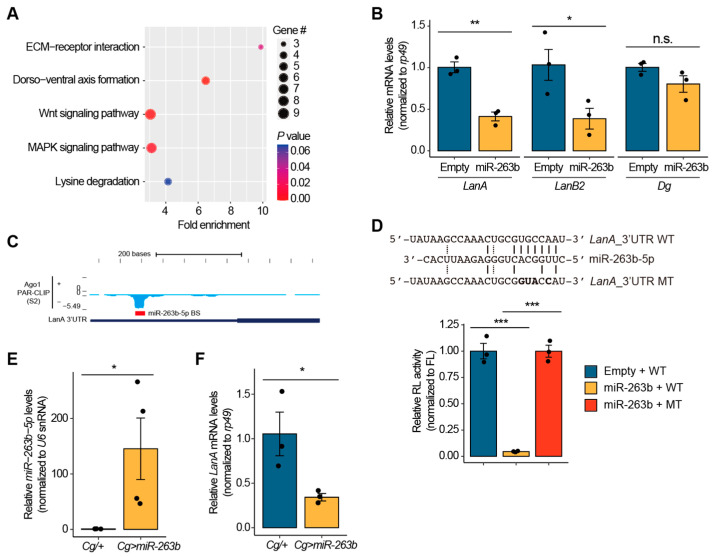
*miR-263b-5p* negatively regulates *LanA* in *Drosophila*. (**A**) GO term enrichment analysis of the potential target genes of *miR-263b-5p* in biological processes. (**B**) Expression levels of *LanA*, *LanB2*, and *Dg* mRNA transcripts in *miR-263b-5p*-overexpressing S2 cells. The levels of *rp49* served as an internal control for mRNAs. (**C**) The RNA reads of Ago1 PAR-CLIP-seq data from S2 cells (blue) and the binding site of miR-263b-5p at the 3′-UTR of *LanA* (miR-263b-5p BS; marked as red line). (**D**) Relative *Renilla*-luciferase (RL) activity containing either the wild type (WT) or mutated (MT) 3′-UTR of *LanA* in S2 cells with or without *miR-263b-5p* overexpression. The sequences of miR-263b-5p, and WT and MT the 3′-UTR of *LanA* are shown (top). The mutated sequences are marked in bold letters. The RL activity was normalized to the firefly luciferase (FL) activity (bottom). *** *p* < 0.001 compared with the control, as assessed by ANOVA followed by a Dunnett’s multiple comparison test. (**E**) Relative expression levels of *miR-263b-5p* in the fat bodies of *Cg>miR-263b* larvae. *U6 snRNA* levels served as an internal control for miRNA. (**F**) Relative expression levels of *LanA* mRNA in the fat bodies of *Cg>miR-263b* larvae. All bar graphs (**B**,**E**,**F**) are shown as the mean ± SEM. n.s., not significant; * *p* < 0.05 and ** *p* < 0.01 compared with control, as assessed by Student’s *t*-test.

To explore the underlying molecular mechanisms regulated by miR-263b-5p, we sought to identify the target genes of miR-263b-5p associated with cell association. Accordingly, we searched for the potential target genes of miR-263b-5p using the TargetScanFly database [29]. This search identified 428 predicted target genes with conserved miR-263b-5p binding sites (Supplementary Table S2). To gain insight into the signaling pathways in which these potential targets were enriched, we performed a functional enrichment analysis using the KEGG pathway. Using this approach, five enriched pathways were identified: ECM–receptor interaction, dorsoventral axis formation, Wnt signaling pathway, MAPK signaling pathway, and lysine degradation (Figure 3A). Notably, among the four significant pathways (*p* < 0.05), the ECM–receptor interaction pathway was the most enriched (Figure 3A). Given the likelihood that ECM–related genes are associated with cell adhesion or dissociation, we focused on three potential target genes, *Dystroglycan* (*Dg*), *Laminin B2* (*LanB2*), and *Laminin A* (*LanA*), belonging to the ECM–receptor interaction pathway. To determine whether miR-263b-5p regulates the transcript levels of these three genes, we assessed their expression in *miR-263b*-overexpressing S2 cells using RT-qPCR. The results showed that the expression of *LanA* and *LanB2* was significantly downregulated in *miR-263b*-overexpressing S2 cells, whereas the expression of *Dg* did not differ between the control and *miR-263b*-overexpressing S2 cells (Figure 3B). These results suggest that at least two ECM–related genes, *LanA* and *LanB2*, are regulated by miR-263b-5p at the transcriptional level.

To investigate the detailed regulatory mechanisms underlying the consequences of *miR-263b* mis-expression, we focused solely on *LanA* as the target gene of miR-263b-5p. To assess the possibility of direct targeting of the *LanA* 3′-UTR by miR-263-5p, we analyzed the Ago1 cross-linking immunoprecipitation (CLIP)-seq data generated from S2 cells [39]. Interestingly, we identified a strong and clear peak in the Ago1 CLIP-seq data at the 3′-UTR of *LanA*, which precisely overlapped with the potential binding site of miR-263-5p (Figure 3C). This finding suggests that the miR-263b-5p incorporated into the Ago1 protein, likely binds to the 3′-UTR of *LanA* in S2 cells. To further validate whether miR-263b-5p regulates the expression of *LanA* by directly binding to the site identified from Ago1 CLIP-seq data, we performed a dual-luciferase reporter assay. We generated luciferase reporters containing the wild type (WT) or a mutated 3′-UTR of *LanA* (Figure 3D, top), and then measured *Renilla* luciferase activity under *miR-263b* overexpression conditions. The result showed a significant decrease in the activity of *Renilla* luciferase bearing WT 3′-UTR of *LanA* when *miR-263b-5p* was overexpressed (Figure 3D, bottom). However, this repression activity of miR-263b-5p on the activity of *Renilla* luciferase bearing 3′-UTR of *LanA* was abolished when its putative binding site in the 3′-UTR of *LanA* was mutated. (Figure 3D, bottom). Collectively, these data suggest that miR-263b-5p suppresses *LanA* expression by directly binding to the 3′-UTR of *LanA*. 

In addition, we investigated whether a regulatory network between miR-263b-5p and *LanA* exists in larval fat bodies. To address this, we first confirmed the overexpression of *miR-263b-5p* in the fat bodies of *Cg>miR-263b* larvae (Figure 3E) and then examined the transcript levels of *LanA*. Consistent with the observations in S2 cells, the fat bodies of *Cg>miR-263b* larvae exhibited a significantly lower expression of *LanA* mRNA transcripts than the control larval fat bodies (Figure 3F). Moreover, the expression of *LanB2* mRNA transcripts was also reduced in the fat bodies of *Cg>miR-263b* larvae compared to the control fat bodies (Supplementary Figure S1). These data provide further evidence that *LanA* and *LanB2* are bona fide target genes of *Drosophila* miR-263b-5p.

### 3.4. Depletion of LanA in the fat Body Results in Defects in the Normal Growth

**Figure 4:**
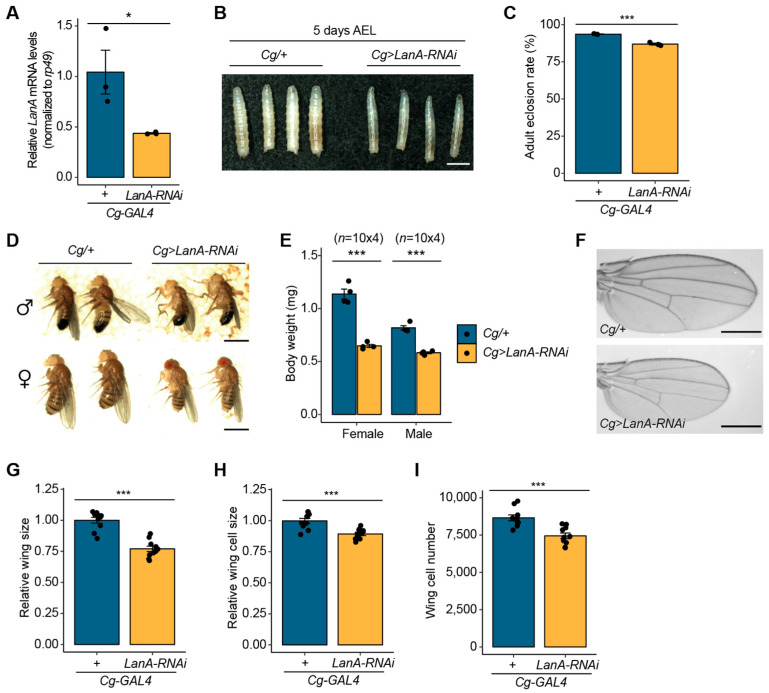
Depletion of *LanA* leads to reduction in developmental growth. (**A**) Relative expression level of *LanA* mRNA transcripts in the fat body of *Cg>LanA-RNAi* larvae. (**B**) Representative images of *Cg>LanA-RNAi* larvae (5 days AEL). Scale bar, 1 mm. (**C**) The effect of *LanA* knockdown in the fat body on eclosion rate. (**D**) Representative images of adult male and female *Cg>LanA-RNAi* flies (3–5 days old). Scale bar, 1 mm. (**E**) Body weight of female and male *Cg>LanA-RNAi* flies (*n* = 10 flies × 4 tubes). (**F**) Representative wing images of adult female flies of the indicated genotype. Scale bar, 0.5 mm. (**G**) Relative wing size of *Cg>LanA-RNAi* adult female flies (*n* = 10 wings). (**H**) Relative wing cell size of *Cg>LanA-RNAi* adult female flies (*n* = 10 wings). (**I)** Total wing cell number of *Cg>LanA-RNAi* adult female flies. *Cg/+* flies were used as the control. All bar plots (**A**,**C**,**E**,**G**,**H**,**I**) are shown as the mean ± SEM. * *p* < 0.05 and *** *p* < 0.001 compared with control, as assessed by Student’s *t*-test.

Given that miR-263b-5p negatively regulates *LanA*, a major component of the BM, we wondered whether a lack of *LanA* expression would lead to consequences similar to those of *miR-263b-5p* overexpression. To knock down *LanA* expression in the fat body, we overexpressed long double-stranded hairpin RNA targeting LanA using *Cg-GAL4* (*Cg>LanA-RNAi*). Through this *UAS-RNAi/GAL4* system, the *LanA* transcript levels effectively decreased in the fat bodies of *Cg>LanA-RNAi* larvae compared to that in the control (Figure 4A). Consistent with the observations in *Cg>miR-263b* larvae, *Cg>LanA-RNAi* larvae at 5 days AEL had smaller body size than *Cg/+* larvae (Figure 4B). Additionally, we monitored the eclosion rate from pupae to adult flies in *Cg>LanA-RNAi*. The depletion of *LanA* in the fat body resulted in a slight reduction of 13.08% in the eclosion rate (Figure 4C). 

Furthermore, adult flies that emerged from *Cg>LanA-RNAi* pupae exhibited a significant decrease in size compared with control flies, regardless of sex (Figure 4D). In line with the reduction in body size, the body weights of both *Cg>LanA-RNAi* female and male flies were dramatically decreased by 56.9 and 71.3%, respectively, compared to control flies (Figure 4E). These observations indicate that *LanA* is involved in normal growth and further support the conclusion that the upregulation of *miR-263b-5p* inhibits developmental growth by targeting *LanA*. 

In addition, we analyzed the wings of *Cg>LanA-RNAi* female adults to assess the changes in cell number and/or size with respect to growth reduction. Consistent with the reduction in body size, the representative wing tissue of *Cg>LanA-RNAi* female adults was significantly reduced relative to that of the controls (Figure 4F,G). We further determined the size and number of wing cells in *Cg>LanA-RNAi* female adults. Both the cell size and number of the *Cg>LanA-RNAi* female adult wings were reduced compared to those of the control wings (Figure 4H,I). These data suggest that the reduced growth of *Cg>LanA-RNAi* adults is associated with a decrease in both cell size and number.

### 3.5. LanA Is Involved in Cell Association in the Fat Body

**Figure 5:**
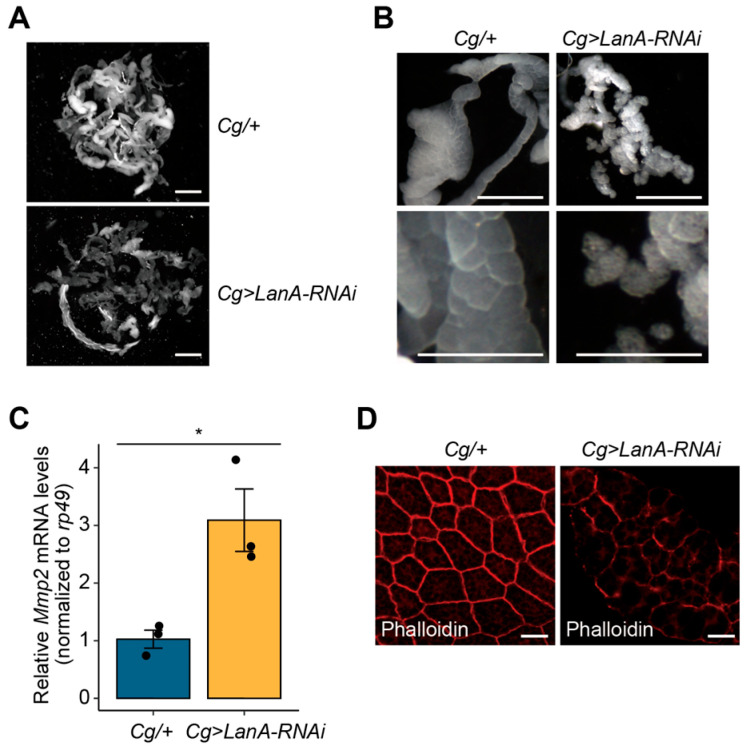
Depletion of *LanA* leads to cell dissociation in the fat body. (**A**) The overall size of the fat bodies dissected from wandering third-instar larvae (WL3) of each genotype (*n* = 5). Scale bar, 1 mm. (**B**) Magnified images of the fat bodies from WL3 of each genotype. Scale bar, 0.5 mm. (**C**) The expression level of *Mmp2* mRNA transcript in the fat body of *Cg> LanA-RNAi* larvae. *rp49* served as an internal control. The bar plot is shown as the mean ± SEM. * *p* < 0.05 compared with control, as assessed by Student’s *t*-test. (**D**) Reduction of F-actin (red) in the fat body of *Cg>LanA-RNAi* larvae. Scale bar, 100 µm.

Next, we wondered whether *LanA*-depletion-caused defects in developmental growth were associated with abnormal cell dissociation in the fat body, as observed from *miR-263b* overexpression. First, we compared the overall size of fat bodies between *Cg/+* and *Cg>LanA-RNAi* larvae. The overall size of the fat bodies was markedly reduced in *Cg>LanA-RNAi* larvae compared to that of the control fat bodies (Figure 5A). We further investigated whether cell dissociation occurred in the reduced fat bodies of *Cg>LanA-RNAi* larvae. Interestingly, similar to the findings for *Cg>miR-263b* larval fat bodies, premature cell dissociation, albeit partial, appeared in the fat bodies of *Cg>LanA-RNAi* larvae (Figure 5B). Taken together, these findings suggest that *LanA* is involved in fat body remodeling. 

Furthermore, we determined the expression levels of *Mmp2* mRNA transcripts and F-actin in the fat bodies of *Cg>LanA-RNAi* larvae. The fat bodies of *Cg>LanA-RNAi* larvae showed a higher expression level of *Mmp2* transcripts than the fat bodies of control larvae (Figure 5C). Additionally, phalloidin-stained F-actin intensity significantly decreased in the *Cg>LanA-RNAi* larval fat body relative to that in the control fat body (Figure 5D). Overall, in the larval fat body, the consequences of *LanA* depletion were similar to the results obtained from miR-263b overexpression. This similarity suggests that the upregulation of *miR-263b* plays a negative role in developmental growth by leading to premature cell dissociation through suppressing ECM–related genes, such as *LanA*.

## 4. Discussion

In this study, we showed that the overexpression of the metamorphosis-associated miRNA, miR-263b, plays a negative role in developmental growth and fat body cell association by suppressing the expression of *LanA*, a major component of the BM in *Drosophila.*

During the larval-to-pupal transition, insects undergo extensive remodeling of various larval tissues, such as the fat body. Part of tissue remodeling entails the degradation of larval tissues through the activation of cell death genes, whereas the larval fat body is remodeled by tissue dissociation [13,40]. Throughout this process, the larval fat body detaches into individual floating fat cells and is redistributed throughout the body of the pupa [13]. The dissociation process can be completed by the destruction of cell–cell junctions, cell–BM junctions, and BM components. As described previously, two *Drosophila Mmps* genes, *Mmp1* and *Mmp2,* are involved in fat body remodeling by inducing degradation of the fat body structure [12]. These *Mmps* are regulated by two main insect hormones: juvenile hormone and 20-hydroxyecdysone [41,42]. Juvenile hormone represses the expression of *Mmps* through the antimetamorphic factor Kr-h1, whereas 20-hydroxyecdysone induces the *Mmps* expression via the DHR3-bftz-F1 axis [41]. Collectively, these two main hormone-signaling pathways regulate *Mmps*, and their induction leads to fat body remodeling by destroying the existing tissue structure. In addition to these mechanisms, our results indicated that the metamorphosis-associated miRNA, miR-263b, can suppress BM formation by directly targeting *LanA* and inducing *Mmp2* in the fat body. However, future studies should investigate whether the expression of miR-263b is regulated by metamorphosis-related insect hormones, and how miR-263b regulates the expression of *Mmp2*. 

According to our results, the consequences of *miR-263b* overexpression were similar to those of *LanA* knockdown. In addition to other in vitro results in support of *LanA* as a target gene of miR-263b-5p, this implies that the phenotypes observed in *Cg>miR-263b* are likely linked to a lack of *LanA*. However, the resulting phenotypes, such as defects in larval growth and dissociation of larval fat body cells in *Cg>miR-263b* seemed more severe than those in *Cg>LanA-RNAi*. Therefore, we cannot rule out the possibility that *miR-263b* might simultaneously regulate the expression of other target genes related to cell dissociation. Indeed, in this study, we revealed that another *Drosophila* laminin-encoding gene, *LanB2* was downregulated by *miR-263b* at the transcript level. Moreover, TargetScanFly predicted many genes implicated in cell adhesion as potential targets of miR-263b-5p, such as *Neuroglian*, *roughest*, *rhea*, *Cadherin 74A*, *Basigin*, *starry night*, *sticks and stones*, *inflated*, *tartan*, *gliolectin*, *off-track*, *Down syndrome cell adhesion molecule 2 and 4*, and *Fasciclin 2* [29]. Thus, complex coordination by miR-263b could lead to a more effective regulation of tissue remodeling. However, to validate our hypothesis, future studies should investigate whether miR-263b regulates these cell adhesion-related genes during tissue remodeling. 

Knockout mutant flies of miR-263b are fertile and viable [21,43], indicating that miR-263b is not essential for the development of adult flies. Nevertheless, our experiments revealed that both *miR-263b* overexpression and *LanA* depletion led to a significant reduction in growth, accompanied by a dramatic decrease in the total fat body mass during development. Therefore, the reduction in developmental growth is likely attributable to growth defects in the fat body, which is crucial for energy metabolism and growth [36]. However, further studies are required to investigate the mechanisms by which miR-263b and *LanA* affect tissue growth. While miR-263b may be directly involved in tissue growth by targeting other genes related to cell size and/or proliferation, the decrease in tissue growth due to *LanA* depletion indicates that ECM might be associated with normal tissue growth. As previously described, various growth factors, such as platelet-derived growth factor (PDGF), fibroblast growth factor 2 (FGF-2), vascular endothelial growth factor (VEGF), and transforming growth factor β1 (TGF-β1), interact with the ECM [44]. The ECM plays a role in reducing the degradation of growth factors and serves as a storage site [44]. Based on these findings, future investigations into the signaling pathways associated with miR-263b and *LanA* will contribute to a more comprehensive understanding of the complex network that regulates tissue remodeling and growth. 

As shown in this study, miR-263b is highly conserved in ecdysozoans. Although information on *LanA* mRNA sequences in other ecdysozoans is limited, the binding sites of miR-263b at the 3′-UTR of *LanA* are well conserved in other *Drosophila* species, such as *Drosophila melanogaster*, *Drosophila pseudoobscura*, and *Drosophila simulans*. This indicates that the regulatory interaction between miR-263b and *LanA* likely plays a crucial role in tissue remodeling and growth across different *Drosophila* species. In conclusion, it appears that the regulatory mechanism of miR-263b/*LanA* involved in tissue remodeling and growth during the transition from larva to pupa in ecdysozoans, which undergo metamorphosis, has been evolutionarily well-conserved. This conservation is likely necessary to simultaneously facilitate the transformation from larval to pupal stages and drive the transition from growth to maturation during development in these species. 

## 5. Conclusions

In this study, we found that untimely overexpression of *miR-263b* in the fat body led to severe defects in developmental growth of *Drosophila*. These defects are associated with premature dissociation of larval fat body cells. Furthermore, we demonstrated that miR-263b-5p negatively regulates the expression of *LanA*, a key component of the BM. The phenotypic consequences of *LanA* depletion were similar to those resulting from *miR-263b* overexpression. Overall, our findings provide valuable insights into the regulatory networks involved in tissue remodeling and developmental growth.

## Data Availability

Datasets are available upon request. The raw data supporting the conclusions of this study will be made available by the authors without any reservations.

## Supplementary Materials

The following supporting information can be downloaded at: www.mdpi.com/xxx/, Supplementary Figure S1: Relative expression levels of *LanA* mRNA in the fat bodies of *Cg>miR-263b* larvae; Supplementary Table S1: Sequences of the oligonucleotides used in this study; Supplementary Table S2: Potential target genes predicted by TargetScanFly.
