# Supplementary material for: MicroRNA miR-263b-5p Regulates Developmental Growth and Cell Association by Suppressing Laminin A in Drosophila
Source: Biology (Basel). 2023 Aug 7;12(8):1096. doi: 10.3390/biology12081096 (PMC10451713; doi:10.3390/biology12081096)
Supplement: Supplementary file 1 [file biology-12-01096-s001.zip › Supplementary_FigureS1.pdf]

Supplementary Figure S1

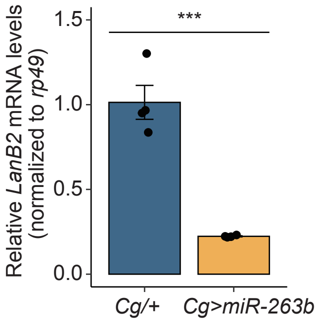

Supplementary Figure S1. Relative expression levels of *LanA* mRNA in the fat bodies of *Cg>miR-263b* larvae. Bar graph is shown as the mean ± SEM. The levels of *rp49* served as an internal control for mRNAs. *** $P < 0.001$ compared with control, as assessed by Student's t-test.
